# Tris(1*H*-benzimidazole-κ*N* ^3^)(pyridine-2,6-dicarb­oxy­lato-κ^3^ *O* ^2^,*N*,*O* ^6^)nickel(II)

**DOI:** 10.1107/S1600536812019514

**Published:** 2012-05-05

**Authors:** Yue-Hua Li, Feng-Feng Li, Xin-Hua Liu, Ling-Yan Zhao

**Affiliations:** a College of Chemical Engineering, Hebei United University, Tangshan 063009, People’s Republic of China; b College of Light Industry, Hebei United University, Tangshan 063009, People’s Republic of China; c Qian’an College, Hebei United University, Tangshan 063009, People’s Republic of China

## Abstract

In the title complex, [Ni(C_7_H_3_NO_4_)(C_7_H_6_N_2_)_3_], the Ni^II^ ion is coordinated by two carboxyl­ate O atoms and the N atom from a pyridine-2,6-dicarboxyl­ate ligand and by three N atoms from three benzimidazole ligands to form a slightly distorted octa­hedral geometry. In the crystal, mol­ecules are linked by N—H⋯O hydrogen bonds to form a three-dimensional network.

## Related literature

For related structures of Ni^II^ dipicolinate complexes, see: Liu *et al.* (2011 ▶).
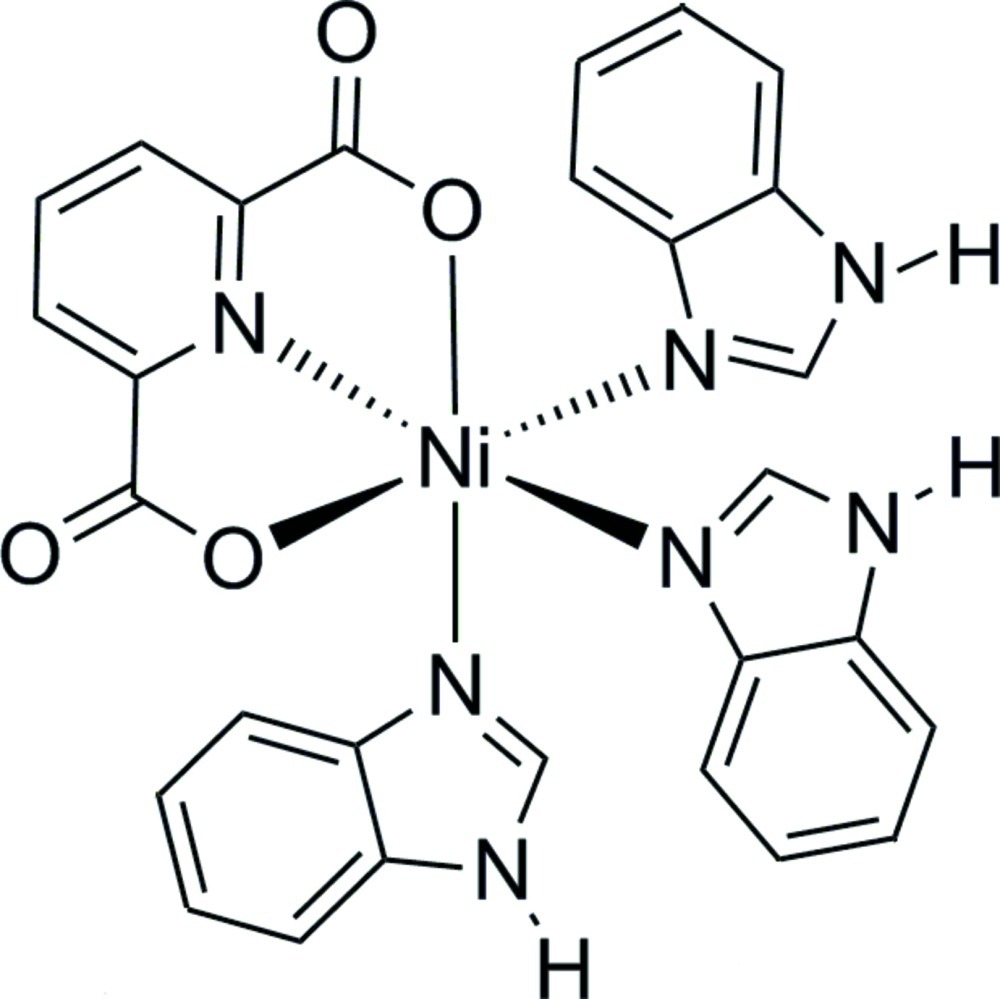


## Experimental

### 

#### Crystal data

[Ni(C_7_H_3_NO_4_)(C_7_H_6_N_2_)_3_]
*M* *_r_* = 578.21
Monoclinic, 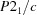

*a* = 9.4546 (19) Å
*b* = 10.487 (2) Å
*c* = 27.532 (5) Å
β = 98.94 (3)°
*V* = 2696.7 (9) Å^3^
*Z* = 4
Mo *K*α radiation
μ = 0.77 mm^−1^
*T* = 298 K
0.10 × 0.10 × 0.10 mm

#### Data collection

Bruker APEXII CCD diffractometer
Absorption correction: multi-scan (*SADABS*; Sheldrick, 1996 ▶) *T*_min_ = 0.934, *T*_max_ = 0.934
21674 measured reflections
4752 independent reflections
2842 reflections with *I* > 2σ(*I*)
*R*_int_ = 0.153

#### Refinement

*R*[*F* ^2^ > 2σ(*F* ^2^)] = 0.086
*wR*(*F* ^2^) = 0.143
*S* = 1.12
4752 reflections
361 parameters
H-atom parameters constrained
Δρ_max_ = 0.33 e Å^−3^
Δρ_min_ = −0.32 e Å^−3^

### 

Data collection: *APEX2* (Bruker, 2007 ▶); cell refinement: *SAINT* (Bruker, 2007 ▶); data reduction: *SAINT*; program(s) used to solve structure: *SHELXS97* (Sheldrick, 2008 ▶); program(s) used to refine structure: *SHELXL97* (Sheldrick, 2008 ▶); molecular graphics: *SHELXTL* (Sheldrick, 2008 ▶); software used to prepare material for publication: *SHELXTL*.

## Supplementary Material

Crystal structure: contains datablock(s) I, global. DOI: 10.1107/S1600536812019514/lh5462sup1.cif

Structure factors: contains datablock(s) I. DOI: 10.1107/S1600536812019514/lh5462Isup2.hkl

Additional supplementary materials:  crystallographic information; 3D view; checkCIF report

## Figures and Tables

**Table 1:** Hydrogen-bond geometry (Å, °)

*D*—H⋯*A*	*D*—H	H⋯*A*	*D*⋯*A*	*D*—H⋯*A*
N2—H2*A*⋯O4^i^	0.86	1.83	2.683 (6)	169
N4—H4*A*⋯O2^ii^	0.86	2.07	2.775 (6)	139
N6—H6*A*⋯O2^iii^	0.86	2.00	2.856 (6)	177

Symmetry codes: (i) 
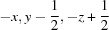
; (ii) 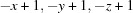; (iii) 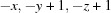.

**Fig. 1.**
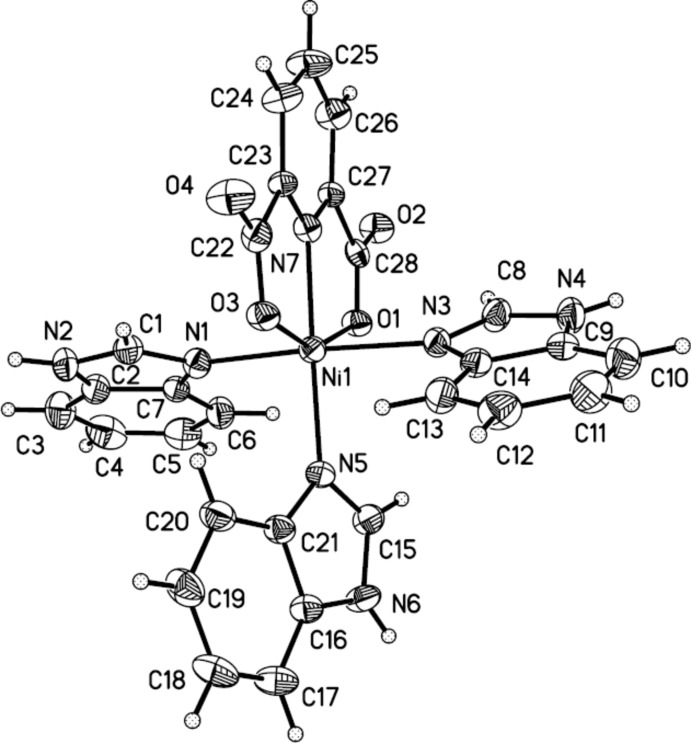
The molecular structure of the title compound with displacement ellipsoids drawn at 30% probability level. H atoms are presented as a small spheres of arbitrary radius.

## supplementary crystallographic information

### Comment

Dipicolinic acid (pyridine–2,6–dicarboxylic acid) has important biological functions in organisms and commonly coordinates to transition metals by either carboxylate bridges between metal centers, to form polymeric or dimeric complexes or tridentate (O, N, O') chelation to one metal ion. Some Ni(II) dipicolinate complexes with imidazoles have been reported (Liu *et al.* 2011). Here, we report here the crystal structure of the title compound.

The molecular structure of the title compound is illustrated in Fig. 1. Bond lengths and angles common to related structures are in the normal range (Liu *et al.* 2011). The Ni^II^ ion is coordinated by two carboxylate oxygen atoms and one N atom from a pyridine-2,6-dicarboxylato ligand and three N atoms from three benzimidazole ligands to form a slightly distorted octahedral geometry. In the crystal, molecules are linked by N—H···O hydrogen bonds to form a three-dimensional network.

### Experimental

Nickel(II) hydroxide (92.7 mg, 1 mmol) was mixed with a 15 ml aqueous solution of dipicoline acid (334 mg, 2 mmol) in a steam bath until the solid disappeared. The methanol solution (10 ml) of benzimidazole (472 mg, 4 mmol) was then added to the above solution. The resultant green solution was warmed on a steam bath for 1.5 h. The solution was filtered and allowed to stand at room temperature. Green crystals suitable for X-ray diffraction were obtained after 15 days.

### Refinement

All H atoms were positioned geometrically an refined using a riding model with C—H = 0.93 Å and *U*_iso_(H) = 1.2*U*_eq_(C) for aromatic H, N—H = 0.86 Å and *U*_iso_(H) = 1.2*U*_eq_(N) for the NH group.

### Crystal data

**Table:**

[Ni(C_7_H_3_NO_4_)(C_7_H_6_N_2_)_3_]	*Z* = 4
*M**_r_* = 578.21	*F*(000) = 1192
Monoclinic, *P*2_1_/*c*	*D*_x_ = 1.424 Mg m^−^^3^
Hall symbol: -P 2ybc	Mo *K*α radiation, λ = 0.71073 Å
*a* = 9.4546 (19) Å	µ = 0.77 mm^−^^1^
*b* = 10.487 (2) Å	*T* = 298 K
*c* = 27.532 (5) Å	Block, green
β = 98.94 (3)°	0.10 × 0.10 × 0.10 mm
*V* = 2696.7 (9) Å^3^	

### Data collection

**Table:**

Bruker APEXII CCD diffractometer	4752 independent reflections
Radiation source: fine-focus sealed tube	2842 reflections with *I* > 2σ(*I*)
Graphite monochromator	*R*_int_ = 0.153
φ and ω scans	θ_max_ = 25.0°, θ_min_ = 3.0°
Absorption correction: multi-scan (*SADABS*; Sheldrick, 1996)	*h* = −11→11
*T*_min_ = 0.934, *T*_max_ = 0.934	*k* = −12→12
21674 measured reflections	*l* = −32→32

### Refinement

**Table:**

Refinement on *F*^2^	Primary atom site location: structure-invariant direct methods
Least-squares matrix: full	Secondary atom site location: difference Fourier map
*R*[*F*^2^ > 2σ(*F*^2^)] = 0.086	Hydrogen site location: inferred from neighbouring sites
*wR*(*F*^2^) = 0.143	H-atom parameters constrained
*S* = 1.12	*w* = 1/[σ^2^(*F*_o_^2^) + (0.0345*P*)^2^ + 1.7256*P*] where *P* = (*F*_o_^2^ + 2*F*_c_^2^)/3
4752 reflections	(Δ/σ)_max_ = 0.004
361 parameters	Δρ_max_ = 0.33 e Å^−^^3^
0 restraints	Δρ_min_ = −0.32 e Å^−^^3^

### Special details

**Table:**

Geometry. All e.s.d.'s (except the e.s.d. in the dihedral angle between two l.s. planes) are estimated using the full covariance matrix. The cell e.s.d.'s are taken into account individually in the estimation of e.s.d.'s in distances, angles and torsion angles; correlations between e.s.d.'s in cell parameters are only used when they are defined by crystal symmetry. An approximate (isotropic) treatment of cell e.s.d.'s is used for estimating e.s.d.'s involving l.s. planes.
Refinement. Refinement of *F*^2^ against ALL reflections. The weighted *R*-factor *wR* and goodness of fit *S* are based on *F*^2^, conventional *R*-factors *R* are based on *F*, with *F* set to zero for negative *F*^2^. The threshold expression of *F*^2^ > σ(*F*^2^) is used only for calculating *R*-factors(gt) *etc*. and is not relevant to the choice of reflections for refinement. *R*-factors based on *F*^2^ are statistically about twice as large as those based on *F*, and *R*- factors based on ALL data will be even larger.

### Fractional atomic coordinates and isotropic or equivalent isotropic displacement parameters (Å^2^)

**Table:**

	*x*	*y*	*z*	*U*_iso_*/*U*_eq_	
Ni1	0.09815 (7)	0.43868 (7)	0.38239 (2)	0.0360 (2)	
O1	0.1890 (4)	0.3672 (3)	0.45593 (12)	0.0401 (10)	
O2	0.3778 (4)	0.2602 (4)	0.49567 (13)	0.0502 (11)	
O4	0.2432 (4)	0.4481 (4)	0.24837 (14)	0.0621 (12)	
N1	−0.0148 (5)	0.2663 (4)	0.36705 (16)	0.0394 (12)	
N7	0.2861 (4)	0.3605 (4)	0.37243 (16)	0.0337 (11)	
O3	0.1016 (4)	0.4723 (3)	0.30609 (12)	0.0415 (10)	
N3	0.2048 (5)	0.6140 (4)	0.40368 (16)	0.0404 (12)	
N5	−0.0917 (4)	0.5276 (4)	0.39039 (17)	0.0407 (12)	
C9	0.3706 (6)	0.3067 (5)	0.4103 (2)	0.0360 (14)	
C10	−0.2085 (6)	0.5587 (5)	0.35471 (19)	0.0393 (14)	
C11	0.3074 (6)	0.3110 (5)	0.4579 (2)	0.0380 (14)	
C12	0.4561 (6)	0.3234 (5)	0.3201 (2)	0.0525 (17)	
H12A	0.4830	0.3293	0.2890	0.063*	
C13	−0.0723 (6)	0.1829 (5)	0.3985 (2)	0.0387 (14)	
N4	0.3530 (5)	0.7511 (5)	0.44750 (18)	0.0543 (14)	
H4A	0.4155	0.7799	0.4710	0.065*	
C15	−0.1244 (6)	0.5731 (6)	0.4318 (2)	0.0471 (16)	
H15A	−0.0643	0.5670	0.4619	0.057*	
C16	0.1950 (6)	0.7325 (5)	0.3796 (2)	0.0415 (15)	
C17	0.2152 (6)	0.4356 (5)	0.2902 (2)	0.0413 (14)	
N2	−0.1251 (5)	0.1077 (5)	0.32270 (18)	0.0532 (14)	
H2A	−0.1576	0.0625	0.2973	0.064*	
C19	−0.1402 (6)	0.0824 (6)	0.3708 (2)	0.0485 (16)	
N6	−0.2532 (5)	0.6289 (4)	0.42619 (17)	0.0494 (13)	
H6A	−0.2931	0.6632	0.4490	0.059*	
C21	0.3245 (5)	0.3690 (5)	0.32835 (19)	0.0325 (13)	
C22	0.5470 (7)	0.2690 (6)	0.3587 (2)	0.064 (2)	
H22A	0.6363	0.2382	0.3542	0.077*	
C23	−0.3100 (6)	0.6213 (5)	0.3772 (2)	0.0418 (15)	
C24	0.1126 (6)	0.7707 (6)	0.3352 (2)	0.0507 (17)	
H24A	0.0502	0.7146	0.3165	0.061*	
C25	0.5027 (6)	0.2609 (5)	0.4046 (2)	0.0508 (17)	
H25A	0.5622	0.2248	0.4311	0.061*	
C26	−0.0736 (6)	0.1876 (6)	0.4489 (2)	0.0518 (17)	
H26A	−0.0298	0.2539	0.4680	0.062*	
C27	−0.3606 (7)	0.5783 (6)	0.2782 (2)	0.0623 (19)	
H27A	−0.3798	0.5641	0.2445	0.075*	
C28	0.2870 (6)	0.8200 (6)	0.4068 (2)	0.0430 (15)	
C29	−0.2074 (7)	−0.0108 (7)	0.4412 (3)	0.075 (2)	
H29A	−0.2512	−0.0755	0.4565	0.089*	
C30	−0.0497 (6)	0.2167 (6)	0.3230 (2)	0.0442 (15)	
H30A	−0.0246	0.2533	0.2948	0.053*	
C31	−0.2331 (6)	0.5352 (6)	0.3045 (2)	0.0522 (17)	
H31A	−0.1664	0.4920	0.2892	0.063*	
C32	−0.1423 (7)	0.0900 (7)	0.4692 (3)	0.065 (2)	
H32A	−0.1455	0.0914	0.5028	0.079*	
C33	0.1278 (7)	0.8935 (7)	0.3208 (2)	0.064 (2)	
H33A	0.0743	0.9215	0.2915	0.077*	
C34	0.3009 (6)	0.6319 (6)	0.4433 (2)	0.0492 (16)	
H34A	0.3298	0.5681	0.4661	0.059*	
C35	0.2207 (8)	0.9783 (7)	0.3484 (3)	0.075 (2)	
H35A	0.2269	1.0612	0.3370	0.090*	
C36	−0.4618 (7)	0.6430 (7)	0.3015 (3)	0.066 (2)	
H36A	−0.5464	0.6714	0.2828	0.079*	
C37	0.3030 (7)	0.9447 (7)	0.3917 (3)	0.069 (2)	
H37A	0.3656	1.0016	0.4099	0.083*	
C38	−0.4384 (6)	0.6650 (6)	0.3507 (2)	0.0562 (18)	
H38A	−0.5055	0.7075	0.3661	0.067*	
C39	−0.2081 (7)	−0.0165 (6)	0.3912 (3)	0.066 (2)	
H39A	−0.2518	−0.0830	0.3722	0.080*	

### Atomic displacement parameters (Å^2^)

**Table:**

	*U*^11^	*U*^22^	*U*^33^	*U*^12^	*U*^13^	*U*^23^
Ni1	0.0339 (4)	0.0399 (4)	0.0320 (4)	0.0057 (4)	−0.0019 (3)	−0.0012 (4)
O1	0.036 (2)	0.052 (3)	0.030 (2)	0.008 (2)	−0.0010 (18)	0.0041 (18)
O2	0.049 (3)	0.065 (3)	0.034 (2)	0.012 (2)	−0.001 (2)	0.011 (2)
O4	0.065 (3)	0.090 (3)	0.033 (2)	0.024 (3)	0.014 (2)	0.018 (2)
N1	0.044 (3)	0.040 (3)	0.032 (3)	0.004 (2)	0.000 (2)	0.000 (2)
N7	0.034 (3)	0.031 (3)	0.034 (3)	0.003 (2)	−0.003 (2)	0.008 (2)
O3	0.035 (2)	0.052 (3)	0.035 (2)	0.0104 (19)	−0.0010 (19)	0.0037 (18)
N3	0.038 (3)	0.038 (3)	0.042 (3)	0.002 (2)	−0.005 (2)	−0.005 (2)
N5	0.034 (3)	0.041 (3)	0.045 (3)	0.006 (2)	−0.001 (2)	−0.003 (2)
C9	0.029 (3)	0.035 (3)	0.042 (4)	0.004 (3)	0.001 (3)	−0.001 (3)
C10	0.036 (3)	0.043 (4)	0.037 (3)	0.004 (3)	0.002 (3)	0.001 (3)
C11	0.035 (4)	0.042 (4)	0.034 (4)	−0.005 (3)	−0.004 (3)	0.001 (3)
C12	0.053 (4)	0.058 (4)	0.051 (4)	0.017 (3)	0.020 (3)	0.008 (3)
C13	0.034 (3)	0.038 (4)	0.042 (4)	0.007 (3)	−0.003 (3)	−0.002 (3)
N4	0.054 (3)	0.049 (4)	0.055 (4)	−0.004 (3)	−0.007 (3)	−0.012 (3)
C15	0.046 (4)	0.056 (4)	0.036 (4)	0.011 (3)	−0.005 (3)	−0.003 (3)
C16	0.037 (4)	0.040 (4)	0.045 (4)	0.010 (3)	0.000 (3)	−0.001 (3)
C17	0.046 (4)	0.039 (4)	0.035 (4)	0.004 (3)	−0.003 (3)	0.000 (3)
N2	0.050 (3)	0.052 (4)	0.053 (4)	−0.004 (3)	−0.004 (3)	−0.018 (3)
C19	0.043 (4)	0.047 (4)	0.053 (4)	0.005 (3)	−0.001 (3)	−0.007 (4)
N6	0.049 (3)	0.057 (3)	0.043 (3)	0.018 (3)	0.010 (3)	−0.005 (3)
C21	0.031 (3)	0.032 (3)	0.034 (3)	0.005 (3)	0.005 (3)	0.003 (3)
C22	0.046 (4)	0.089 (6)	0.058 (5)	0.032 (4)	0.009 (4)	0.014 (4)
C23	0.040 (4)	0.045 (4)	0.041 (4)	0.008 (3)	0.006 (3)	0.006 (3)
C24	0.052 (4)	0.044 (4)	0.051 (4)	0.005 (3)	−0.003 (3)	−0.001 (3)
C25	0.048 (4)	0.058 (4)	0.044 (4)	0.015 (3)	−0.001 (3)	0.009 (3)
C26	0.052 (4)	0.050 (4)	0.053 (4)	−0.006 (3)	0.007 (3)	−0.005 (3)
C27	0.049 (4)	0.094 (6)	0.041 (4)	0.001 (4)	−0.002 (3)	0.001 (4)
C28	0.034 (3)	0.043 (4)	0.051 (4)	0.005 (3)	0.003 (3)	−0.002 (3)
C29	0.062 (5)	0.066 (6)	0.097 (7)	−0.003 (4)	0.015 (5)	0.027 (5)
C30	0.043 (4)	0.049 (4)	0.039 (4)	0.004 (3)	0.005 (3)	−0.002 (3)
C31	0.038 (4)	0.071 (5)	0.047 (4)	0.007 (3)	0.003 (3)	−0.003 (3)
C32	0.062 (5)	0.069 (6)	0.066 (5)	0.007 (4)	0.013 (4)	0.010 (4)
C33	0.066 (5)	0.063 (5)	0.061 (5)	0.017 (4)	0.001 (4)	0.008 (4)
C34	0.054 (4)	0.039 (4)	0.051 (4)	0.006 (3)	−0.003 (3)	−0.004 (3)
C35	0.076 (5)	0.046 (5)	0.100 (6)	0.006 (4)	0.007 (5)	0.011 (4)
C36	0.037 (4)	0.092 (6)	0.065 (5)	0.011 (4)	−0.001 (4)	0.021 (4)
C37	0.068 (5)	0.045 (5)	0.092 (6)	−0.004 (4)	0.005 (4)	0.000 (4)
C38	0.048 (4)	0.068 (5)	0.053 (4)	0.017 (4)	0.011 (4)	0.017 (4)
C39	0.069 (5)	0.044 (5)	0.085 (6)	−0.013 (4)	0.010 (5)	−0.003 (4)

### Geometric parameters (Å, º)

**Table:**

Ni1—N7	2.014 (4)	N2—C30	1.347 (7)
Ni1—N5	2.065 (4)	N2—C19	1.379 (7)
Ni1—N1	2.109 (4)	N2—H2A	0.8600
Ni1—N3	2.134 (4)	C19—C39	1.384 (8)
Ni1—O3	2.135 (3)	N6—C23	1.375 (6)
Ni1—O1	2.204 (3)	N6—H6A	0.8600
O1—C11	1.259 (6)	C22—C25	1.393 (7)
O2—C11	1.262 (6)	C22—H22A	0.9300
O4—C17	1.227 (6)	C23—C38	1.393 (7)
N1—C30	1.314 (6)	C24—C33	1.362 (8)
N1—C13	1.398 (7)	C24—H24A	0.9300
N7—C21	1.323 (6)	C25—H25A	0.9300
N7—C9	1.337 (6)	C26—C32	1.377 (8)
O3—C17	1.281 (6)	C26—H26A	0.9300
N3—C34	1.320 (6)	C27—C31	1.382 (7)
N3—C16	1.405 (6)	C27—C36	1.406 (8)
N5—C15	1.317 (6)	C27—H27A	0.9300
N5—C10	1.398 (6)	C28—C37	1.387 (8)
C9—C25	1.370 (7)	C29—C39	1.377 (8)
C9—C11	1.523 (7)	C29—C32	1.395 (9)
C10—C23	1.386 (7)	C29—H29A	0.9300
C10—C31	1.387 (7)	C30—H30A	0.9300
C12—C22	1.383 (7)	C31—H31A	0.9300
C12—C21	1.385 (7)	C32—H32A	0.9300
C12—H12A	0.9300	C33—C35	1.391 (9)
C13—C26	1.390 (7)	C33—H33A	0.9300
C13—C19	1.397 (7)	C34—H34A	0.9300
N4—C34	1.342 (7)	C35—C37	1.364 (8)
N4—C28	1.397 (7)	C35—H35A	0.9300
N4—H4A	0.8600	C36—C38	1.359 (7)
C15—N6	1.339 (6)	C36—H36A	0.9300
C15—H15A	0.9300	C37—H37A	0.9300
C16—C24	1.402 (7)	C38—H38A	0.9300
C16—C28	1.398 (7)	C39—H39A	0.9300
C17—C21	1.524 (7)		
			
N7—Ni1—N5	176.78 (18)	C39—C19—C13	123.1 (6)
N7—Ni1—N1	93.10 (17)	C15—N6—C23	107.3 (5)
N5—Ni1—N1	89.25 (17)	C15—N6—H6A	126.3
N7—Ni1—N3	89.93 (16)	C23—N6—H6A	126.3
N5—Ni1—N3	87.88 (17)	N7—C21—C12	120.7 (5)
N1—Ni1—N3	175.03 (18)	N7—C21—C17	114.2 (5)
N7—Ni1—O3	77.57 (16)	C12—C21—C17	125.1 (5)
N5—Ni1—O3	100.17 (16)	C12—C22—C25	119.0 (6)
N1—Ni1—O3	91.68 (15)	C12—C22—H22A	120.5
N3—Ni1—O3	92.82 (16)	C25—C22—H22A	120.5
N7—Ni1—O1	76.15 (16)	N6—C23—C10	105.9 (5)
N5—Ni1—O1	106.06 (16)	N6—C23—C38	132.1 (6)
N1—Ni1—O1	90.31 (15)	C10—C23—C38	122.0 (5)
N3—Ni1—O1	86.59 (16)	C33—C24—C16	117.0 (6)
O3—Ni1—O1	153.72 (14)	C33—C24—H24A	121.5
C11—O1—Ni1	114.6 (3)	C16—C24—H24A	121.5
C30—N1—C13	104.9 (5)	C9—C25—C22	119.4 (5)
C30—N1—Ni1	124.8 (4)	C9—C25—H25A	120.3
C13—N1—Ni1	130.3 (4)	C22—C25—H25A	120.3
C21—N7—C9	121.9 (5)	C32—C26—C13	117.2 (6)
C21—N7—Ni1	118.1 (3)	C32—C26—H26A	121.4
C9—N7—Ni1	119.9 (4)	C13—C26—H26A	121.4
C17—O3—Ni1	115.4 (3)	C31—C27—C36	121.2 (6)
C34—N3—C16	104.4 (5)	C31—C27—H27A	119.4
C34—N3—Ni1	125.8 (4)	C36—C27—H27A	119.4
C16—N3—Ni1	129.7 (4)	C37—C28—C16	123.1 (6)
C15—N5—C10	105.0 (4)	C37—C28—N4	132.2 (6)
C15—N5—Ni1	125.5 (4)	C16—C28—N4	104.7 (5)
C10—N5—Ni1	129.4 (4)	C39—C29—C32	121.3 (7)
N7—C9—C25	120.2 (5)	C39—C29—H29A	119.4
N7—C9—C11	113.1 (5)	C32—C29—H29A	119.4
C25—C9—C11	126.6 (5)	N1—C30—N2	113.5 (5)
C23—C10—C31	120.5 (5)	N1—C30—H30A	123.3
C23—C10—N5	108.9 (5)	N2—C30—H30A	123.3
C31—C10—N5	130.6 (5)	C27—C31—C10	117.5 (6)
O2—C11—O1	125.9 (5)	C27—C31—H31A	121.2
O2—C11—C9	118.0 (5)	C10—C31—H31A	121.2
O1—C11—C9	116.2 (5)	C26—C32—C29	122.3 (7)
C22—C12—C21	118.8 (6)	C26—C32—H32A	118.9
C22—C12—H12A	120.6	C29—C32—H32A	118.9
C21—C12—H12A	120.6	C24—C33—C35	122.1 (6)
C26—C13—C19	119.8 (6)	C24—C33—H33A	118.9
C26—C13—N1	131.2 (5)	C35—C33—H33A	118.9
C19—C13—N1	109.0 (5)	N3—C34—N4	113.6 (5)
C34—N4—C28	107.5 (5)	N3—C34—H34A	123.2
C34—N4—H4A	126.3	N4—C34—H34A	123.2
C28—N4—H4A	126.3	C37—C35—C33	122.6 (7)
N5—C15—N6	113.0 (5)	C37—C35—H35A	118.7
N5—C15—H15A	123.5	C33—C35—H35A	118.7
N6—C15—H15A	123.5	C38—C36—C27	121.3 (6)
C24—C16—C28	119.6 (6)	C38—C36—H36A	119.4
C24—C16—N3	130.6 (5)	C27—C36—H36A	119.4
C28—C16—N3	109.8 (5)	C35—C37—C28	115.5 (6)
O4—C17—O3	127.1 (5)	C35—C37—H37A	122.2
O4—C17—C21	118.3 (5)	C28—C37—H37A	122.2
O3—C17—C21	114.6 (5)	C36—C38—C23	117.4 (6)
C30—N2—C19	106.9 (5)	C36—C38—H38A	121.3
C30—N2—H2A	126.5	C23—C38—H38A	121.3
C19—N2—H2A	126.5	C29—C39—C19	116.3 (6)
N2—C19—C39	131.1 (6)	C29—C39—H39A	121.9
N2—C19—C13	105.7 (5)	C19—C39—H39A	121.9

### Hydrogen-bond geometry (Å, º)

**Table:**

*D*—H···*A*	*D*—H	H···*A*	*D*···*A*	*D*—H···*A*
N2—H2*A*···O4^i^	0.86	1.83	2.683 (6)	169
N4—H4*A*···O2^ii^	0.86	2.07	2.775 (6)	139
N6—H6*A*···O2^iii^	0.86	2.00	2.856 (6)	177

Symmetry codes: (i) −*x*, *y*−1/2, −*z*+1/2; (ii) −*x*+1, −*y*+1, −*z*+1; (iii) −*x*, −*y*+1, −*z*+1.
